# Bridged repair of large ventral hernia defects using an ovine reinforced biologic: A case series

**DOI:** 10.1016/j.amsu.2022.103446

**Published:** 2022-03-02

**Authors:** George DeNoto

**Affiliations:** General Surgery Department, St. Francis Hospital, Roslyn, NY, 11576, USA

**Keywords:** Case series, Reinforced tissue matrix, Biologic matrices, Ovine reinforced biologic, Large ventral hernia, Bridged repair

## Abstract

**Introduction:**

Of all hernia types, large ventral hernias have the most impact on patient quality of life, however they are also the most difficult type of hernia to repair and are associated with high rates of complications. This case series describes repair of large ventral hernias with an ovine reinforced biologic in a complex patient cohort with comorbidities and concomitant procedures.

**Methods:**

The author performed bridged repair with an ovine reinforced biologic in 19 consecutive high-risk patients over a 5-year period. In all cases the reinforced biologic was used as an underlay.

**Outcomes:**

Of the 19 patients, six (32%) experienced a surgical site occurrence including infection, seroma, abscess, fistula, bioloma, or bowel obstruction. Three patients (16%) had recurrences with two out of three of the recurrences occurring within 6 months of surgery.

**Conclusions:**

Rates of SSO's and recurrences using ovine reinforced tissue matrix (RTM) were in line with or better than other published studies of bridged repair utilizing biologic or synthetic mesh reinforcement. Ovine RTM's should therefore be considered in complex large ventral hernia repairs.

## Highlights

• Large ventral hernias greatly impact patient quality of life.
• In some cases, large ventral hernias necessitate bridged repair with mesh.
• 19 patients received bridged repair with an ovine reinforced matrix.
• Mean follow up of 23 months showed low complication and recurrence rates.

## 1 Introduction

In the United States, abdominal wall or ventral hernias are common with a prevalence of 1.7% for the population as a whole [1]. Ventral hernias vary in size and severity. Large ventral hernias (≥8–10 cm width or loss of domain) [[2], [3], [4]] have the greatest impact on patient quality of life, with patients reporting major physical, psychological, and social problems [3]. Large ventral hernias are also the most challenging for surgeons to repair and are associated with high rates of surgical site occurrences (SSO's) [2,3]. Both surgeon and patient factors influence the outcome of large ventral hernia repairs [5]. While some factors can be controlled for, others cannot. Factors that can be controlled include the surgeon's choice of technique and any materials used in an effort to encourage wound healing and prevent recurrence.

Large ventral hernias can be successfully repaired using either intraoperative components separation with primary fascial closure or bridged repair [2]. Components separation attempts to allow re-approximation of the abdominal fascia at the defect site to achieve primary closure [2]. Botulinum toxin, or Botox, is commonly used preoperatively with components separation to increase musculofascial advancement and promote closure [6]. However, not all hernia defects can be closed primarily even when using preoperative Botox and intraoperative components separation. In these instances, bridged repair must be performed in which mesh is placed as a bridge posterior to the remaining fascial gap [2]. This mesh must therefore provide high biomechanical strength to bear the load of the abdominal wall forces [7].

Inclusion of mesh in hernia repairs has resulted in decreased recurrences compared to suturing alone [8,9]. For this reason, almost all hernia repairs conducted today utilize a mesh reinforcement. Traditional meshes are composed of synthetic polymers, however, some studies have shown that use of these synthetic meshes correlates with surgical site infections (SSI's) and the need for explantation [10,11]. These issues led to the use of biologic meshes composed of the extracellular matrices (ECMs) from animal or human tissues. While smaller ventral hernias with lower risk of SSO's may be successfully treated with synthetic mesh, surgeons may choose a biologic mesh for large ventral hernia repair as these patients are already at high risk of post-operative infections [3]. Unfortunately, due to their propensity to stretch, certain biologic meshes such as human acellular dermal matrices (ADMs) are not always the best choice for large ventral hernia repair despite their association with lower rates of SSO's than synthetic meshes [12]. Because no ideal synthetic or biologic product has been identified for use in complex large ventral hernia procedures, some surgeons have begun using hybrid reinforced biologic meshes for this application.

In this case series, our tertiary referral center for complex abdominal wall reconstruction used an ovine reinforced biologic in the repair of large ventral hernias requiring bridging. Specifically, we chose OviTex® 1S and 2S Permanent (P) products (TELA Bio Inc., Malvern, PA, USA) which combine 6 or 8 layers of decellularized ovine ECM, respectively, with 4% polypropylene polymer fiber interwoven through the layers in a lockstitch pattern for reinforcement. These products were used in the bridged repair of large ventral hernias in 19 patients with a high incidence of comorbidities and prior ventral hernia recurrences, between 2016 and 2021. In this first case series of its kind, the use of this ovine reinforced biologic resulted in lower complication and recurrence rates in this complex patient population compared to the author's previous experience with other mesh types and published case series of bridged patients utilizing other mesh types [[13], [14], [15]].

## 2 Material and methods

### 2.1 Study design

Nineteen patients who underwent large ventral hernia bridged repair by the author (G.D.) from November 2016 to June 2021 at St. Francis Hospital were included in this case series. The author is the chief of general surgery at St. Francis Hospital, has been practicing for 28 years, and performs over 400 hernia repairs a year. St. Francis Hospital in Rosyln, NY, USA is a community hospital which is staffed by medical students and physician's assistants. The study was conducted as a retrospective, single-center, consecutively enrolled study. Patients were not formally recruited and instead retrospectively identified. An IRB waiver of consent from St. Francis Hospital Institutional Review Board (IRB) (IRB # – 21–46) was obtained due to the retrospective nature of the study (IRB # – 21–46 accessible at: https://www.researchregistry.com/register-now#user-researchregistry/registerresearchdetails/61e5d28175d23b002014734e/UIN: 7563). OviTex 1S-Permanent or 2S-Permanent (TELA Bio Inc., Malvern PA) was used in all patients to perform the bridged repair. Patient demographics, preoperative variables, and comorbid conditions were collected for each patient. All patient level data was deidentified by removal of specified individual identifiers prior to analysis. Data collection was ongoing between November 2016 to December 2021. Data analysis was performed between August 2021 and December 2021. This case series has been reported in line with the PROCESS 2020 (www.processguideline.com) criteria [16].

### 2.2 Inclusion criteria

Patients with a history of wound infection or at high risk of developing a post-operative wound infection were included in this study. Patients included in this study required a bridged hernia repair at surgery when it was determined intraoperatively the fascia could not be reapproximated for primary closure. These patients were also noted to be classified on the Ventral Hernia Working Group (VHWG) scale as VHWG grade 2 and grade 3 with complicating factors including, but not limited to obesity, hypertension, and previous abdominal surgery.

### 2.3 Exclusion criteria

Patients who did not have a large ventral hernia necessitating bridged repair. Patients with a ventral hernia classified as grade 1 or grade 4 on the Ventral Hernia Working Group (VHWG) scale or patients who had a VHWG grade 2 hernia but did not have other complicating factors.

### 2.4 Pre-intervention patient optimization

Prior to surgery, patients were requested to attain certain goals or adhere to specific protocols depending on their comorbid conditions. Four of the patients in this case series were emergent and were not able to participate in pre-operative optimization. Diabetic patients were required to have hemoglobin A1C levels less than 6.5. Efforts were made in obese patients to lose weight, if possible, to obtain a BMI less than 40. Patients who currently smoked were asked to quit. A pre-op bacterial decontamination protocol was prescribed for the seven patients who were at highest infection risk to reduce the risk of post-operative surgical site infections. The bacterial decontamination protocol consisted of applying Bactroban ointment twice per day tothe nares for 5 days prior to surgery. The bacterial decontamination protocol also involved daily chlorohexidine soap showers for 5 days prior to surgery. A pre-habilitation exercise regimen was also recommended for 4–6 weeks prior to surgery as these regimens have been shown to reduce length of hospital stay in patients undergoing thoracic surgery [17]. In addition, all elective patients were treated with our enhanced recovery after surgery (ERAS) protocol which have also been found affective in decreasing length of hospital stay by addressing pain control and prioritizing GI function [18]. According to this protocol, patients were administered Tylenol, NSAIDs, oxycodone, and gabapentin pre-operatively. Post-operatively patients were administered analgesics including oxycodone, gabapentin, and muscle relaxants such as valium. Post-operative pain management combined with early feeding is intended to reduce post-operative ileus.

### 2.5 Surgical method

Surgical methodology was standardized as much as possible given patient comorbidities and concomitant surgeries. A specific preoperative hernia characterization protocol was not utilized as the author has determined that protocols designed to measure hernia sac volume and % loss of domain[19] do not take into consideration the compliance of the abdominal wall. Through his extensive experience, the author has found the “Kocher test” described below to be the most valuable tool to characterize each specific hernia. All surgeries involved excision of old scar tissue, skin, and the subcutaneous tissue of the abdominal wall in the location of the hernia sac. If possible, peri-umbilical perforators, branches of the inferior epigastric artery, were preserved in an effort to maintain good perfusion of skin and subcutaneous tissues to minimize the risk of wound ischemia and infection. Next the abdominal contents were reduced back into the peritoneal cavity. Fascial approximation was then assessed using the “Kocher test.” The Kocher test was performed by placing 3 Kocher clamps on either side of the fascial edges and pulling them together to assess fascial closure. When the Kocher test failed and the fascia was unable to be brought together the patient was deemed to meet the criteria for components separation. At this point the type of components separation to be performed was decided. If the defect was found to be less than 10 cm during the Kocher test, a posterior components separation transversus abdominus release was chosen. If the defect was greater than 10 cm with a non-compliant abdominal wall, then an anterior components separation external oblique release was chosen as it gives more release and advancement of the fascia than does a posterior release. In all cases, mesh was placed as a wide underlay. Mesh was secured at least 5 cm lateral to the bridged area using full thickness sutures through the abdominal wall musculature with interrupted U suture placement. A second row of sutures were placed in a running fashion suturing the fascial edge to the top few layers of the without full thickness to avoid deep injury. In the majority of cases, prolene sutures were utilized, however in some cases PDS sutures were utilized. In all cases, drains were placed subcutaneously. A gentamycin or clindamycin antibiotic rinse was utilized in all cases. A Prevena® Incision VAC (3 M, Saint Paul, Minnesota, USA) was used in some cases as required.

### 2.6 Follow up

Post-operative follow up was performed for all patients via office visit with some long term follow up via telephone consultation. Mean follow up for all patients was 23 months with a range from 5 to 61 months after discharge. Follow up is ongoing.

### 2.7 Endpoints

Endpoints for this study included hernia recurrence, surgical site occurrences (SSO's), and adverse events. SSO's consisted of surgical site infections, wound dehiscence, seromas, abscesses, fistulas, biloma, and bowel obstructions. SSO's, adverse events, and recurrences were detected during post-operative physical examinations that were scheduled between 2 weeks and 6 months post-surgery. Patients also scheduled additional follow-up appointments as necessary to address specific concerns. Hernia recurrences were diagnosed by physical examination and sometimes diagnosed by CT scan.

## 3 Results

A consecutive cohort of 19 patients who had large hernia defects repaired using OviTex 1S–P or 2S–P were included in this case series. These patients had a high incidence of comorbidities and a history of prior ventral hernia recurrences (Table 1). These comorbidities were significant and included a 58% rate of obesity, a 42% rate of hypertension, and a 21% rate of cancer (Table 1). The patient population had a mean age of 59 and was composed of more females than males (11 females and 8 males) (Table 1). No deviations from the planned surgical procedure occurred. No patients were lost to follow up. All patients adhered to surgeon post-operative recommendations, such as rehab, as assessed at follow up via office visit or telephone conversation.

**Table 1:** Patient demographics, preoperative variables, comorbid conditions.

Subjects Enrolled – 19	
Sex, n (%)	Female: 11 (58%), Male: 8 (42%)
Age (years), mean ± SEM (range)	59.1 ± 2.86, (24–78)
Body Mass Index (kg/m^2^), mean ± SEM (range)	33.6 ± 2.13, (23–35.4)
Comorbidities, n (%)	Hypertension: 8 (42%), Obesity: 11 (58%), Crohn's/Colitis: 2 (11%), Prior or current smoker: 2 (11%), Coronary Artery Disease: 2 (11%), Hyperthyroidism: 2 (11%), Hyperlipidemia: 4 (21%), Diabetes Mellitus: 4 (21%), Factor V Leiden: 1 (5%), Pulmonary Hypertension: 2 (11%), Asthma: 2 (11%), Diverticulitis: 1 (5%), Cancer: 4 (21%), End Stage Renal Disease: 1 (5%), Gout: 1 (5%), Gallbladder disease: 1 (5%), Congestive Heart Failure: 1 (5%), Lupus: 1 (5%), Stroke: 1 (5%), Parkinson's 1 (5%)
Patients with Prior VH repairs, n (%)	13 (68%)
Patients with Prior VH Mesh Repairs, n (%)	9 (69%) out of 13 prior VH repairs
Prior non-VH surgery, n (%)	16 (84%)
Prior SSI, n (%)	6 (32%)

**Fig. 1:**
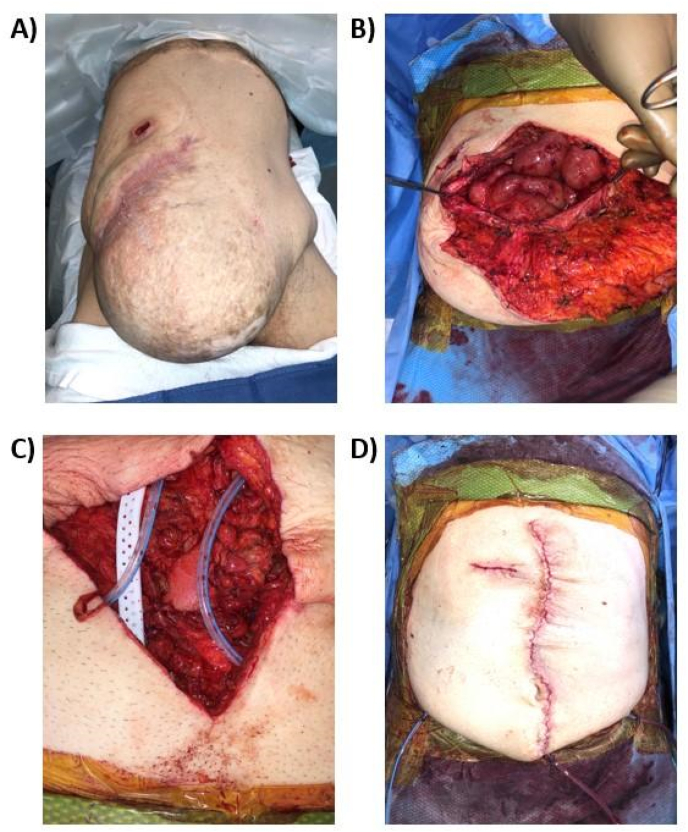
Bridged repair of large ventral hernia using OviTex RTM. A) Hernia pre-operation. B) Dissection of hernia sac. C) Placement of drains subcutaneously. D) Bridged closure of defect.

Bridged repair was the only option to treat these large ventral defects present in this patient population, where primary fascial closure was not possible (Fig. 1). The average hernia defect size in this cohort was 12 × 18 cm with a range from 6 × 10 cm to 15 × 27 cm (Table 2). These hernias were complicated as 37% were classified as a grade 2 (comorbid) on the VHWG scale and 63% were classified as a grade 3 (potentially contaminated) (Table 2). To achieve closure, these defects required an average bridge size of 4 × 10 cm with a range from 2 × 4 cm to 8 × 15 cm (Table 2). Mesh sizes ranged from 120 to 750 cm^2^ (mean 437 cm^2^). Due to the severity of these defects, OviTex 2S–P, which contains the maximum 8 layers of decellularized ovine forestomach matrix and a permanent polymer support, was utilized in the majority of patients (68%). Using this technique and reinforcement, skin flap closure was achieved in 100% of the patients. On average, patients were discharged seven days after bridged repair; this average discharge was expected due to the complex nature of these hernias and high rate of comorbidities.

**Table 2:** Operative characteristics.

VHWG Grade, n (%)	**Grade 1:** 0 (0%), **Grade 2:** 7 (37%), **Grade 3:** 12 (63%), **Grade 4:** 0 (0%)
Hernia Defect Size (cm), mean (range)	12x18 (6x10–15x27)
Mesh Size (cm), mean (range)	19x23 (10x12–25x30)
Bridge Size (cm), mean (range)	4x10 (2x4 – 8x15)
Type of OviTex, n (%)	**1S–P:** 6 (32%), **2S–P:** 13 (68%)
Release, n (%)	**Anterior:** 15 (80%), **Posterior:** 4 (21%)
Plane of Placement, n	**Retrorectus:** 4, **Intraperitoneal:** 15
Concomitant Surgery, n (%)	9 (47%)
Post-Operative Discharge (day), mean (range)	7 (4-12)

Bridged repair of large ventral hernias with an ovine reinforced biologic resulted in relatively low rates of adverse events including surgical site occurrences (SSO) and recurrences. Precautionary measures were taken to avoid adverse events in those patients with the most severe hernias. For instance, in especially high-risk patients a Prevena VAC was placed to provide negative pressure. Drains were also placed in patients with abscesses to prevent seroma formation. Despite these precautions, six patients (32%) developed a SSO, including a seroma (5%), a fistula (5%), and five surgical site infections (26%) (Table 3). Using R software (The R Foundation, Vienna, Austria) to run a point biserial correlation, there was a positive correlation between the VHWG grade and the chance of experiencing an SSO, as expected (cor = 0.5, p = 0.02). Three patients (16%) had recurrences, of which two of these patients were diagnosed at their 6 month follow up (Table 3). The third patient developed a recurrent hernia 9 months after their bridged repair (Table 3). The three patients who developed recurrences were all obese with BMI's of 39, 50, and 55 kg/m^2^. Using the point biserial correlation test mentioned above, there was a strong positive correlation between BMI and recurrence, meaning the higher the patient BMI the higher the chance of recurrence (cor = 0.7, p = 0.0007). These three patients were also all hypertensive and over 55 years of age. None of the recurrent patients developed a post-operative wound infection and there were no infections nor removals of the reinforced biologic itself. The mean follow up for this cohort was 23 months (Table 3). These outcomes are in line with or better than metanalysis results of bridged repair cases with either biologic or synthetic mesh in a similarly complex patient population citing an average 45.8% SSO rate, 29.2% SSI rate, and 25% recurrence rate with a slightly shorter follow up time (average = 16 months) [2].

**Table 3:** Primary and secondary endpoints: Adverse events.

Average Follow Up, range	23 months, (5–61 months)
Hernia Recurrence, n (%)	3 (16%)
Occurrence of an SSO^a^, n (%)	6 (32%)
Seroma, n (%)	1 (5%)
Abscess, n (%)	1 (5%)
Biloma, n (%)	1 (5%)
Fistula, n (%)	1 (5%)
Wound Dehiscence, n (%)	1 (5%)
Surgical Site Infection, n (%)	5 (26%)
Bowel Obstruction, n (%)	1 (5%)
Pulmonary Embolism, n (%)	1 (5%)

a Individual Patients may have experienced more than one SSO.

Many of the patients (13, 68%) in this cohort had previous ventral hernia repairs (Table 1). The majority (9 out of 13) of these previous repairs were completed with a mesh reinforcement (Table 1). Of the previous repairs, 5 were synthetic and 4 were biologic meshes. Prior synthetic mesh repairs led to infection in 3/5 patients and bowel adhesions in 2/5 of patients. Two patients had two recurrences with synthetic meshes before repair with OviTex reinforced biologic. One patient had two recurrences with porcine ADM's and had to have MRSA infected mesh removed, but has remained recurrence and complication free for 16 months after bridged repair with OviTex. Only one of the patients who received previous mesh repair had a recurrence after use of OviTex, which was diagnosed at their 6-month follow-up. The recurrence rate with an ovine reinforced biologic in this patient subset (1/13, 7.7%) is therefore lower than expected when considering these patients' previous recurrences with synthetic and traditional biologic meshes.

## 4 Discussion

This case series is the first of its kind to show the utility of using OviTex reinforced biologic for bridged repair of large, complex ventral hernias in patients with multiple comorbidities where fascial approximation is not possible. The rates of surgical site infections (SSI's) found in this cohort were in line with or better than studies of other bridged repair cohorts that utilized mesh [2,14]. Despite 63% of the patient population having a potentially contaminated defect, the rate of surgical site infection (SSI) was only 26%. This rate of SSI's is lower than the findings of Patel et al. who saw an SSI rate of 44% in a population of patients who underwent bridged repair with a porcine acellular dermal matrix (PADM) [14]. The population in Patel et al. consisted of 33% of patients with comorbid hernias (VHWG grade 2), 22% of patients with potentially contaminated hernias (VHWG grade 3) and 44% of patients with infected hernias (VHWG grade 4) compared to our 37% of patients with comorbid hernias with other complicating factors and our 63% of patients with potentially contaminated hernias [14]. The patient cohort in the Patel et al. study had a similar mean follow up time of 18 months compared to the mean of 23 months in this series [14]. The 26% SSI rate in this series was also close to that of a metanalysis conducted by Holihan et al. which found that 29% of patients who received a bridged repair with either synthetic or biologic mesh experienced an SSI [2]. The population in Holihan et al. consisted of 30.8% of patients with potentially contaminated and 15.4% with infected hernias compared to our 63% of patients with potentially contaminated hernias [2]. The follow up time for the patient population in Holihan et al. was also comparable with a median of 16 months compared to the median follow up of 19 months [2].

Rate of recurrence was also in line with or better than studies which utilized other types of mesh to perform bridged repair [[2], [13], [14], [15], [20]]. A recurrence rate of 16% was observed in this cohort, with 2/3 recurrences occurring by the 6 month follow up. In comparison, a previous study that the senior author participated in found a recurrence rate of 44% when patients with complex hernias were bridged with a porcine acellular dermal matrix (PADM) [20]. The follow up time for this study (24 months) was similar to the mean follow up time of 23 months in this series [20]. Other studies have even higher rates of recurrence with other mesh types. For instance, Patel et al. and Abdelfatah et al. found that 89% and 80% of patients, respectively, had recurrences when PADM was used in bridged repair [14,15]. While the Patel et al. study had a similar median follow up time as mentioned above, the Abdelfatah et al. study followed all patients to 60 months [14,15]. Blatnik et al. found a similar rate of recurrence as Patel et al. and Abdelfatah et al. (80%) with a human ADM with a mean follow up time nearly identical to this study [13]. A metanalysis of bridged cases in which synthetic mesh was used 36% of the time and biologic mesh used 64% of the time found an average recurrence rate of 25%; more similar to our findings in this study [2]. This metanalysis performed by Holihan et al. had a similar median follow up time as detailed above [2]. The low recurrence rate in this series was also notable due to the complexity of the patient population and the severity of their BMI's with an average of 34 kg/m^2^. In this study the rate of recurrence was positively correlated with BMI (cor = 0.7, p = 0.0007) providing evidence that BMI does in fact directly affect recurrence rate.

The three obese recurrent patients are currently awaiting recurrent incisional hernia repair surgery. These three patients are all taking measures to reduce their BMI's in hopes of having better surgical outcomes. One patient is attempting self weight loss, but is considering bariatric surgery. A second patient had a bariatric procedure after their initial hernia repair and has lost 110 lbs, attaining a BMI of 39 compared to their original BMI of 55 at initial surgery. This patient plans on a combined hernia repair and panniculectomy. The third patient has been successful on their preoperative exercise regimen and has reduced their BMI from 39 to 35, however, she continues to work on smoking cessation prior to proceeding with recurrent hernia repair surgery.

The strengths of this study are that it is homogenous in several regards. It only included patients with severe, large hernia defects that required bridging. All procedures were performed by the same surgical team. These similarities remove certain between-subject differences that would otherwise convolute the true effect that repair with a reinforced biologic had on the outcomes. However, the study is not without its weaknesses and limitations. This study was limited by a lack of control group(s). In addition, the sample size was relatively small and follow up was not regimented to specific timepoints. The surgical technique was also not completely standardized.

This is the first publication specifically showing the performance of a reinforced biologic matrix in bridged hernia repair. The outcomes of this study demonstrate that use of a reinforced biologic in this complex hernia repair patient population is safe and effective. The use of a reinforced biologic matrix may lead to equivalent or lower rates of SSI's and recurrences than other mesh options in bridged repair. These equivalent or lower rates are present despite a similar high risk patient population. Future studies are necessary to make more definitive comparisons than to those results in the literature. A randomized controlled trial in which high-risk patients receive a synthetic, traditional biologic, or reinforced biologic matrix would help to make more definitive comparisons among the three different matrix types. Pre- and post-operative standardization in such a trial would also help to remove any compounding factors.

## 5 Statements and disclosures

A waiver of informed consent was obtained for publication of this case series and accompanying images. The waiver of informed consent is available upon request.

This study was approved by St. Francis Hospital Institutional Review Board (IRB) IRB # – 21–46.

This case series was presented as a poster at the Society of American Gastrointestinal and Endoscopic Surgeons (SAGES) conference August 31-September 2, 2021.

Medical writing assistance was provided by TELA Bio Inc.

## Provenance and peer review

Not commissioned, externally peer-reviewed.

## Funding

Funding for the manuscript submission fee was provided by TELA Bio Inc.

## Ethical approval

St. Francis Hospital in Roslyn New York approved this study IRB # – 21–46.

## Please state any sources of funding for your research

Funding for researchregistry.com and the manuscript submission fee was provided by TELA Bio Inc.

Medical writing assistance was provided by TELA Bio Inc.

Please specify the contribution of each author to the paper, e.g. study concept or design, data collection, data analysis or interpretation, writing the paper, others, who have contributed in other ways should be listed as contributors.

G.D. study concept and design, data collection, data analysis and interpretation, writing the paper.

Medical writing assistance was provided by TELA Bio Inc.

## Please state any conflicts of interest

G.D. is a consultant for TELA Bio Inc.

## Registration of research studies

1. Name of the registry: researchregistry.com
2. Unique Identifying number or registration ID: researchregistry7563
3. Hyperlink to your specific registration (must be publicly accessible and will be checked): https://www.researchregistry.com/register-now#user-researchregistry/registerresearchdetails/61e5d28175d23b002014734e/

## Guarantor

Dr. George DeNoto III.

## Consent

A waiver of informed consent was obtained for publication of this case series and accompanying images. All patient information has been deidentified to ensure privacy. A copy of the IRB waiver is available for Review by the Editor-in-Chief of this journal on request.

## Annals of medicine and surgery

The following information is required for submission. Please note that failure to respond to these questions/statements will mean your submission will be returned. If you have nothing to declare in any of these categories then this should be stated.

## Declaration of competing interest

G.D. is a consultant for TELA Bio Inc.

## References

[1] Beadles C.A., Meagher A.D., Charles A.G. (2015). Trends in emergent hernia repair in the United States. Jama Surg..

[2] Holihan J.L., Askenasy E.P., Greenberg J.A., Keith J.N., Martindale R.G., Roth J.S. (2016). Component separation vs. Bridged repair for large ventral hernias: a multi-institutional risk-adjusted comparison, systematic review, and meta-analysis. Surg. Infect..

[3] Thomsen C.Ø., Brøndum T.L., Jørgensen L.N. (2016). Quality of life after ventral hernia repair with endoscopic component separation technique. Scand. J. Surg..

[4] Carbajo M.A., Olmo JCM del, Blanco J.I., Cuesta C de la, Martín F., Toledano M. (2000). Laparoscopic treatment of ventral abdominal wall hernias: preliminary results in 100 patients. J. Soc. Laparoendosc. Surg..

[5] Faylona J.M. (2017). Evolution of ventral hernia repair. Asian J. Endosc. Surg..

[6] Bueno-Lledó J., Martinez-Hoed J., Torregrosa-Gallud A., Menéndez-Jiménez M., Pous-Serrano S. (2020). Botulinum toxin to avoid component separation in midline large hernias. Surgery.

[7] Boukovalas S., Sisk G., Selber J.C. (2018). Abdominal wall reconstruction: an integrated approach. Semin. Plast. Surg..

[8] Burger J.W.A., Luijendijk R.W., Hop W.C.J., Halm J.A., Verdaasdonk E.G.G., Jeekel J. (2004 Oct). Long-term follow-up of a randomized controlled trial of suture versus mesh repair of incisional hernia. Ann. Surg..

[9] Luijendijk R.W., Hop W.C., Tol MP van den, Lange DC de, Braaksma M.M., IJzermans J.N. (2000 Aug 10). A comparison of suture repair with mesh repair for incisional hernia. N. Engl. J. Med..

[10] Kokotovic D., Bisgaard T., Helgstrand F. (2016 Oct 18). Long-term recurrence and complications associated with elective incisional hernia repair. JAMA.

[11] Hawn M.T., Gray S.H., Snyder C.W., Graham L.A., Finan K.R., Vick C.C. (2011 Jul). Predictors of mesh explantation after incisional hernia repair. Am. J. Surg..

[12] Moya MA de, Dunham M., Inaba K., Bahouth H., Alam H.B., Sultan B. (2008). Long-term outcome of acellular dermal matrix when used for large traumatic open abdomen. J. Trauma Inj. Infect. Crit. Care.

[13] Blatnik J., Jin J., Rosen M. (2008 Jul). Abdominal hernia repair with bridging acellular dermal matrix--an expensive hernia sac. Am. J. Surg..

[14] Patel K.M., Nahabedian M.Y., Albino F., Bhanot P. (2013 Feb). The use of porcine acellular dermal matrix in a bridge technique for complex abdominal wall reconstruction: an outcome analysis. Am. J. Surg..

[15] Abdelfatah M.M., Rostambeigi N., Podgaetz E., Sarr M.G. (2015). Long-term outcomes (>5-year follow-up) with porcine acellular dermal matrix (Permacol^TM^) in incisional hernias at risk for infection. Hernia.

[16] Agha R.A., Sohrabi C., Mathew G., Franchi T., Kerwan A., O'Neill N. (2020). The PROCESS 2020 guideline: updating consensus preferred reporting of CasE series in surgery (PROCESS) guidelines. Int. J. Surg..

[17] Sanchez-Lorente D., Navarro-Ripoll R., Guzman R., Moises J., Gimeno E., Boada M. (2018). Prehabilitation in thoracic surgery. J. Thorac. Dis..

[18] Stearns E., Plymale M.A., Davenport D.L., Totten C., Carmichael S.P., Tancula C.S. (2018). Early outcomes of an enhanced recovery protocol for open repair of ventral hernia. Surg. Endosc..

[19] Winters H., Knaapen L., Buyne O.R., Hummelink S., Ulrich D.J.O., Goor H van (2019). Pre-operative CT scan measurements for predicting complications in patients undergoing complex ventral hernia repair using the component separation technique. Hernia.

[20] Itani K.M.F., MR M.D., DJV M.D., SSA M.D., GDI M.D., CEB M.D. (2012 Sep). Prospective study of single-stage repair of contaminated hernias using a biologic porcine tissue matrix: the RICH Study. Surgery.

